# Clinical predictors of early surgical intervention in patients with venomous snakebites

**DOI:** 10.1186/s40001-023-01101-x

**Published:** 2023-03-21

**Authors:** Hsiao-Yu Lu, Yan-Chiao Mao, Po-Yu Liu, Kuo-Lung Lai, Cheng-Yeu Wu, Yueh-Chi Tsai, Jung-Hsing Yen, I.-Chen Chen, Chih-Sheng Lai

**Affiliations:** 1 Department of Orthopedic, Taichung Veterans General Hospital, Taichung, Taiwan, Republic of China; 2 Division of Clinical Toxicology, Department of Emergency Medicine, Taichung Veterans General Hospital, Taichung, Republic of China; 3 Division of Clinical Toxicology and Occupational Medicine, Department of Medicine, Taipei Veterans General Hospital, Taipei, Taiwan, Republic of China; 4 School of Medicine, National Defense Medical Center, Taipei, Taiwan, Republic of China; 5 Institute of Environmental and Occupational Health Sciences, School of Medicine, National Yang-Ming University, Taipei, Taiwan, Republic of China; 6 Division of Infection, Department of Internal Medicine, Taichung Veterans General Hospital, Taichung, Taiwan, Republic of China; 7 Rong Hsing Research Center for Translational Medicine, National Chung Hsing University, Taichung, Taiwan, Republic of China; 8 Division of Allergy, Immunology and Rheumatology, Department of Internal Medicine, Taichung Veterans General Hospital, Taichung, Taiwan, Republic of China; 9 Division of Plastic and Reconstructive Surgery, Department of Surgery, Taichung Veterans General Hospital, Taichung, Taiwan, Republic of China; 10 Department of Post‐Baccalaureate Medicine, College of Medicine, National Chung Hsing University, Taichung, Taiwan, Republic of China

**Keywords:** Early surgical intervention, Venomous snakebite, *Naja atra*, Antivenom, Envenomation

## Abstract

**Background:**

Venomous snakebites induce tissue destruction and secondary infection; however, the optimal timing of surgical intervention for these complications remains unknown. This study assessed the clinical predictors of early surgical intervention in patients with snakebites.

**Methods:**

This retrospective study included 63 patients (45 men and 18 women) with venomous snakebites. In addition to the snake species, the demographics, affected body parts, clinical characteristics, and ultrasound findings of the patients in the surgical (32 patients) and nonsurgical (31 patients) groups were analyzed and compared.

**Results:**

A higher incidence of acute compartment syndrome, local ecchymosis, skin necrosis, bullae, blisters, and fever was found in the surgical group than in the nonsurgical group, and ultrasound findings of the absence of Doppler flow were more frequently noted in the surgical group than in the nonsurgical group. After adjustment using a multivariate logistic regression model, only advanced age, *Naja atra* bite, local ecchymosis, and bulla or blister formation remained significant factors for surgical intervention. Furthermore, comparison of the outcomes of patients who received early (≤ 24 h) and late (> 24 h) surgical intervention revealed that the duration of continuous negative pressure wound therapy (6 vs. 15 days; *P* = 0.006), duration of hospital stay (13 vs. 26 days; *P* = 0.002), and duration of outpatient follow-up (15 vs. 36 days; *P* < 0.001) were significantly lower in patients who received early surgical intervention. The final reconstructive surgery was simple among the patients who received surgical intervention within 24 h of being bitten (*P* = 0*.*028).

**Conclusion:**

In patients with snakebites, advanced age, high-risk clinical manifestations (e.g., local ecchymosis and bulla or blister formation), and *Naja atra* envenomation are predictors of surgical intervention within 24 h.

## Background

Many snake varieties thrive in Taiwan’s subtropical climate. Six major venomous snakes—*Naja atra* (Chinese cobra), *Bungarus multicinctus* (Taiwanese krait), *Protobothrops mucrosquamatus* (brown-spotted pit viper), *Trimeresurus stejnegeri* (green bamboo viper), *Deinagkistrodon acutus* (sharp-nosed pit viper), and *Daboia siamensis* (Siamese Russell’s viper)—are found throughout Taiwan [1]. Snakebites are a major public health concern, with the nationwide annual incidence of snake bites being 40.49 per million people in Taiwan [1]. According to the World Health Organization’s 2016 guidelines for the management of snakebites, antivenom administration is the most essential treatment strategy for venomous snakebites [2–6].

Although the systemic treatment of patients with venomous snakebites is essential, local treatment cannot be neglected. Local necrotic tissue debridement or finger or toe amputation can cause disability. Herzel et al. [7] reported a 25% amputation rate among patients with severe snakebites. Despite proper treatment, venomous snakebites can cause death or the loss of a body part, thereby affecting patients’ quality of life.

Indications for surgical intervention in snakebite are fairly clear, although the timing of intervention remains subject to debate. Mao et al. [8] have advocated surgical intervention for patients with wound necrosis, abscess formation, gangrene in digits, and necrotizing fasciitis. Su et al. [9] reported that Taiwanese patients with *Naja atra* envenomation who present with skin ecchymosis or require a high antivenom dose should be evaluated to determine the requirement of immediate surgery. *Naja atra*, *Protobothrops mucrosquamatus*, and *Trimeresurus stejnegeri* bites mainly induce local tissue injuries [10–12]. A neurotoxic effect is temporary or absent in *Naja atra* bites [10]. Although some studies have recommended the surgical removal of snake venom as the immediate treatment approach [9, 13–17], other studies have indicated that this approach may cause soft tissue damage, leading to a failed skin graft or flap, amputation, or osteomyelitis and ultimately resulting in poor prognosis [6, 18, 19]. Thus, the recommended protocol for venomous snakebites is the administration of antivenom, followed by delayed debridement [3, 6, 18]. Moreover, Cumpston [19] did not recommend surgical intervention for patients with Crotalinae envenomation. The requirement and optimal timing of surgery for venomous snakebite cases remain controversial. If the requirement of surgery in such cases can be predicted, interventions such as fasciotomy, dermotomy, fasciectomy, and debridement can be performed promptly, thereby reducing the risks of tissue damage and comorbidity.

In this study, we assessed the snake species, clinical symptoms, ultrasound findings, and timing and incidence of surgical intervention to determine the clinical predictors of surgical intervention in venomous snakebite cases.

## Methods

In this retrospective study, the electronic medical records of patients with venomous snakebites who received only medical (antivenom or antibiotic) treatment or a combination of medical and surgical treatment between January 2016 and March 2022 at Taichung Veterans General Hospital were reviewed. Patients who were ≥ 20 years old and were hospitalized due to snakebites were included in this study. Patients who underwent surgery unrelated to the bite site area were excluded. The snake species were identified after examining patients with snakebites who were brought to the emergency department (ED) or by asking the patient to identify the snake in a picture shown to them in the ED. Patients with snakebites for which the culprit snake could not be identified were included in either the other or negative identification group.

The patients were divided into two groups: surgical and nonsurgical groups. In addition to the snake species, the patients’ demographics, bitten body parts, clinical characteristics, and findings of ultrasound imaging of the bite site performed within 24 h were compared. The six P’s were used to diagnose acute compartment syndrome related to snakebites: pain, paresthesia, pallor, paralysis, poikilothermia, and pulselessness [3, 20, 21]. If the patients presented with any one of these symptoms, they were considered to have impending compartment syndrome. If the patients presented with more than two of these symptoms, they were highly suspected of having acute compartment syndrome. Fasciotomy or dermotomy was indicated if acute compartment syndrome was suspected at the bite site. Ultrasound imaging of the bite site was performed within 24 h by one of the authors (K-L Lai) by using a 12-MHz linear array probe. The imaging was performed to identify the location of tissue edema and the presence or absence of Doppler flow. The surgical group was further divided into two subgroups: one subgroup that underwent surgery within 24 h of being bitten and the other underwent subgroup that underwent surgery after 24 h. The wounds of the patients in both the subgroups were postoperatively treated with negative pressure wound therapy (NPWT), and their dressing foams were changed twice per week. After surgical intervention, bite wounds were treated with NPWT immediately after the signs of toxicity and infection spread subsided. The snake species; patients’ demographics, bitten body parts, and clinical characteristics; total antivenom dose; timing of surgical intervention; number of debridements; duration of dressing changes following NPWT; requirement of final reconstructive surgery; duration of hospital stay; and follow-up periods until complete wound healing were compared between the subgroups.

The clinical data and outcomes are summarized as frequencies and percentages. The chi-squared test, Fisher’s exact test, or the Mann–Whitney U test was performed to determine the associations between baseline parameters and surgical intervention outcomes. A *P* value of < 0.05 was considered significant. Univariate and multivariate logistic regression analyses were conducted to analyze the factors significantly associated with surgical intervention, and odds ratios and relevant 95% confidence intervals were calculated. All data were analyzed using SPSS version 22.0 (2013 release; IBM Corp., Armonk, NY, USA). This study was approved by the Institutional Review Board of Taichung Veterans General Hospital (Approval Number CE21125A).

## Results

**Fig. 1:**
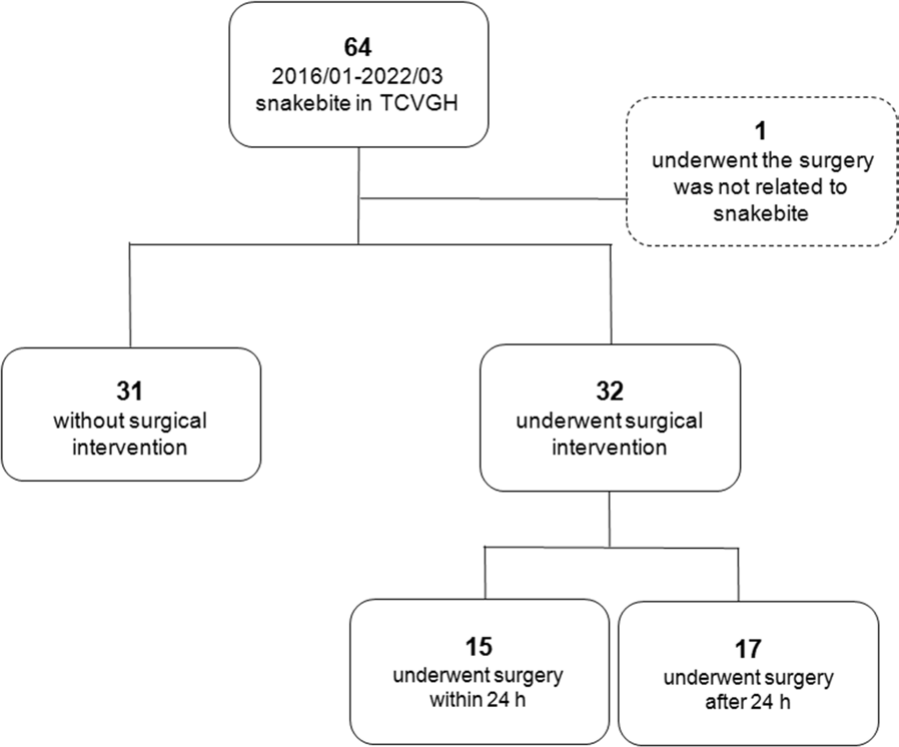
Of the included patients, 32 received surgical intervention and 31did not. TCVGH: Taichung Veterans General Hospital

From January 2016 to March 2022, a total of 64 patients presented with venomous snakebites. One of these patients was excluded because he underwent surgery that was unrelated to the snakebite area. Of the 63 included patients, 31 patients had relatively mild symptoms of toxicity, did not require surgery, and were administered antivenom and hospitalized for empiric antibiotic therapy, symptom relief, wound care, and vital sign monitoring. They were discharged as soon as their wound became smooth and their vital signs were stable. No patient in the nonsurgical group required rehospitalization or surgical intervention. In total, 32 patients with severe local symptoms required surgical intervention to alleviate tissue swelling, control infection, and clean necrotic debris (Fig. 1).

**Fig. 2:**
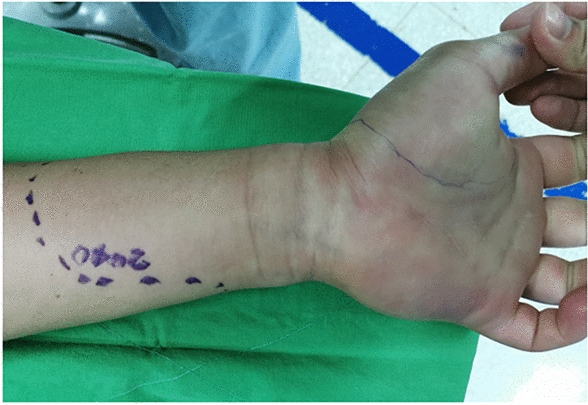
Acute compartment syndrome of the left hand 8 h after being bitten by *Naja atra*

**Fig. 3:**
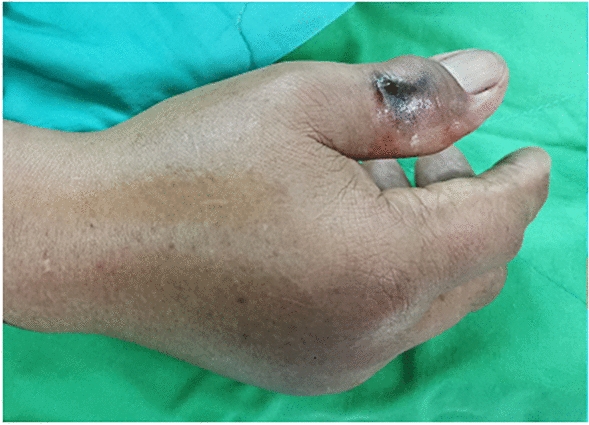
Local ecchymosis on the right thumb 10 h after being bitten by *Naja atra*

**Fig. 4:**
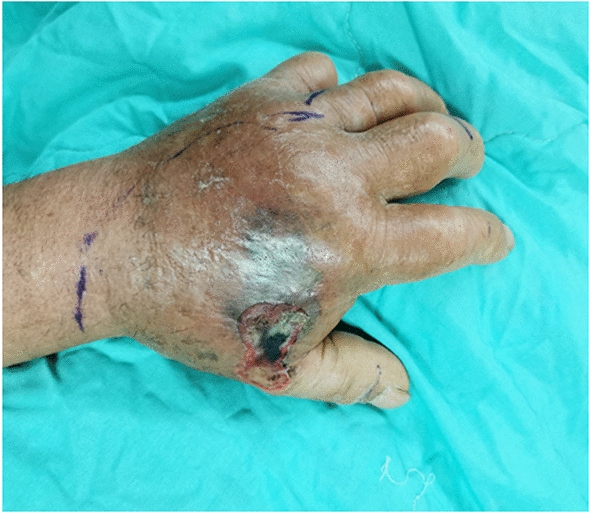
Skin necrosis on the left dorsal hand 48 h after being bitten by *Naja atra*

**Fig. 5:**
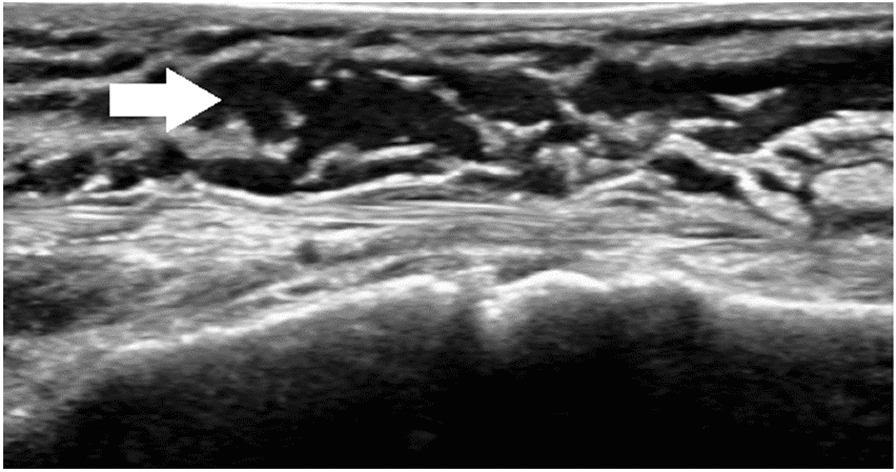
Subcutaneous interstitial edema (white arrow) and absence of Doppler flow were noted on ultrasound

This study included 45 men and 18 women, with both surgical and nonsurgical groups predominantly consisting of men (62.5% and 80.6%, respectively; *P* = 0.111). Overall, 59.4% of the patients in the surgical group had been bitten by *Naja atra*. By contrast, *Trimeresurus stejnegeri* bites were predominant (32.3%) in the nonsurgical group (*P* < 0.001). Acute compartment syndrome (Fig. 2) was more highly suspected in the surgical group than in the nonsurgical group (34.4% vs. 3.2%; *P* = 0.002). Local ecchymosis (Fig. 3; 87.5% vs. 51.6%; *P* = 0.002); skin necrosis (Fig. 4; 28.1% vs.3.2%; *P* = 0.013); bullae or blisters (56.3% vs.9.7%; *P* < 0.001); fever with a temperature of ≥ 38 °C,as measured using a tympanic thermometer (31.2% vs.3.2%; *P* = 0.003); and positive ultrasound findings of absence of Doppler flow (Fig. 5; 68.8% vs.0%; *P* < 0.001) were more commonly noted in the surgical group than in the nonsurgical group (Table 1). No patient required admission to the intensive care unit (ICU), ventilator support, or inotropic support; developed systemic bleeding; or died during the study period.

**Table 1:** Characteristics and clinical manifestations of 63 patients with snakebites

Characteristic data	Operated cases (n = 32)	Non-operated cases (n = 31)	p value
Male, n (%)	20 (62.5%)	25 (80.6%)	0.111^a^
Age (years), median (range)	59 (24–90)	49 (11–84)	0.052^c^
Body part bitten, n			0.513^a^
Upper limb (finger)	17 (12)	19 (13)	
Lower limb (toe)	15 (7)	12 (3)	
Venomous snake, n (%)			< 0.001^a^
*Naja* *atra*	19 (59.4%)	3 (9.7%)	
*Protobothrops* *mucrosquamatus*	5 (15.4%)	8 (25.8%)	
*Trimeresurus* *stejnegeri*	4 (7.7%)	10 (32.3%)	
*Bungarus* *multicinctus*	0	6 (19.4%)	
Others or negative identification	4 (12.5%)	4 (12.9%)	
Swelling, n (%)	30 (93.8%)	26 (83.9%)	0.257^b^
Acute compartment syndrome, suspected, n (%)	11 (34.4%)	1 (3.2%)	0.002^b^
Local ecchymosis, n (%)	28 (87.5%)	16 (51.6%)	0.002^a^
Skin necrosis, n (%)	9 (28.1%)	1 (3.2%)	0.013^b^
Bullae/blister, n (%)	18 (56.3%)	3 (9.7%)	< 0.001^a^
Numbness, n (%)	1 (3.1%)	4 (12.9%)	0.196^b^
Fever (≥ 38 °C), n (%)	10 (31.2%)	1 (3.2%)	0.003^b^
Positive ultrasound exam^d^	22 (68.8%)	0	< 0.001^a^

^a^Chi-squared test

^b^Fisher’s exact test

^c^Mann–Whitney *U* test

^d^Absence of Doppler flow

To identify the factors associated with surgical intervention, snakebite cases were included in regression analyses. The results of univariate logistic regression analysis revealed that advanced age, *Naja atra* bite, suspicion of acute compartment syndrome, local ecchymosis, skin necrosis, bulla or blister formation, and fever were significantly associated with surgical intervention (Table 2). Furthermore, the results of multivariate logistic regression analysis performed using a forward stepwise selection model revealed that only advanced age, *Naja atra* bite, local ecchymosis, and bulla or blister formation were significantly associated with surgical intervention.

**Table 2:** Univariateand multivariate (through forward stepwise selection) logistic regression analysis of factors associated with surgical intervention in 63 patients with snakebites

	Univariate			Multivariable		
	Odds ratio	95%CI	*P* value	Odds ratio	95%CI	*P* value
Sex						
Female	Reference					
Male	0.40	(0.13–1.25)	0.116			
Age	1.03	(1.00–1.06)	0.032*	1.06	(1.01–1.12)	0.015*
Body part bitten						
Upper limb (Finger)	Reference					
Lower limb (Toe)	1.40	(0.51–3.81)	0.513			
Venomous snake						
*Naja* *atra*	Reference					
*Protobothrops* *mucrosquamatus*	0.10	(0.02–0.52)	0.006**	0.22	(0.03–1.81)	0.161
*Trimeresurusstejnegeri*/*Bungarus* multicinctus	0.04	(0.01–0.20)	< 0.001**	0.06	(0.01–0.55)	0.012*
Others or negative identification	0.16	(0.02–1.00)	0.050	0.23	(0.02–2.36)	0.214
Swelling	2.88	(0.52–16.14)	0.228			
Acute compartment syndrome, suspected	15.71	(1.88–131.14)	0.011*			
Local ecchymosis	6.56	(1.86–23.19)	0.003**	6.84	(1.19–39.44)	0.031*
Skin necrosis	11.74	(1.39–99.40)	0.024*			
Bullae/blister	12.00	(3.02–47.72)	< 0.001**	10.17	(1.53–67.63)	0.016*
Numbness	0.22	(0.02–2.07)	0.184			
Fever (≥ 38 °C)	13.64	(1.62–114.52)	0.016*	4.91	(0.28–87.01)	0.278
Positive ultrasound exam	–					

Logistic regression. *P < 0.05, **P < 0.01

Upper limb bites (66.7% vs. 29.4%; *P* = 0.035) and suspicion of acute compartment syndrome (80% vs. 11.8%; *P* < 0.001) were significantly more common among the patients who underwent initial surgery within 24 h of being bitten than among those who underwent initial surgery after 24 h. The venomous snake species and other clinical manifestations (including local ecchymosis, bulla or blister formation, fever, and positive bacterial wound culture) did not differ significantly between the surgical subgroups (Table 3). The median number of days till the initial surgery in the patients who underwent surgery within and after 24 h of being bitten was 0.5 days (interquartile range [IQR], 0.5–1.0 days) and 7 days (IQR, 1.5–15.0 days), respectively. The median number of times an operation for debridement was performed (2 vs.4 times; *P* = 0.012), median number of days between the application of NPWT dressing foams(6 vs. 15 days; *P* = 0.006), median duration of hospital stay (13 vs. 26 days; *P* = 0.002), and median period of outpatient follow-up until complete wound healing (15 vs. 36 days; *P* < 0.001) were significantly lower among the patients who underwent initial surgery within 24 h of being bitten than among those who underwent initial surgery after 24 h (Table 4). The final reconstructive wound closure surgery was simpler for the within 24 h group than for the later than 24 h group, except for one patient who required a free flap due to severe skin necrosis with tendon exposure (*P* = 0.028).

**Table 3:** Characteristics and clinical manifestations of 32 patients who underwent surgery for snakebites

Characteristic data	Operated cases within 24 h (n = 15)	Operated cases later than 24 h (n = 17)	p value
Male, n (%)	11 (73.3%)	9 (52.9%)	0.234^b^
Age (years), median (range)	56 (24–85)	59 (32–90)	0.406^c^
Body part bitten, n			0.035^b^
Upper limb (Finger)	10 (6)	5 (5)	
Lower limb (Toe)	5 (1)	12 (6)	
Venomous snake, n (%)			0.894^a^
Naja atra	9 (60%)	11 (64.7%)	
Protobothrops mucrosquamatus	3 (20%)	2 (11.8%)	
Trimeresurus stejnegeri	1 (6.7%)	2 (11.8%)	
Others or negative identification	2 (13.3%)	2 (11.8%)	
Acute compartment syndrome, suspected, n (%)	12 (80%)	2 (11.8%)	< 0.001^b^
Local ecchymosis, n (%)	12 (80%)	14 (82.4%)	1.000^b^
Bullae/blister, n (%)	8 (53.3%)	10 (58.8%)	0.755^b^
Fever (≥ 38 °C), n (%)	5 (33.3%)	5 (29.4%)	1.000^b^
Wound with positive bacterial culture, n (%)	9 (60%)	12 (70.1%)	0.529^b^

h: hour

^a^Chi-squared test

^b^Fisher’s exact test

^c^Mann–Whitney *U* test

**Table 4:** Surgical management and outcomes of 32 patients who underwent surgery for snakebites

Outcomes	Operated cases within 24 h (n = 15)	Operated cases later than 24 h (n = 17)	p value
Total antivenom dose in vials, median (IQR)	5 (0–17)	6 (3–18)	0.529^c^
After bite to first surgery in days, median (IQR)	0.5 (0.5–1.0)	5.0 (1.5–15.0)	< 0.001^c^
Times of operation for debridement, median (IQR)	2 (0–5)	4 (1–10)	0.012^c^
Days of application NPWT dressing, median (IQR)	6 (2–15)	15 (3–42)	0.006^c^
Toe or finger amputation, n (%)	2 (13.3%)	4 (23.5%)	0.002^b^
Final reconstructive surgery, n (%)			0.028^a^
Secondary healing	1 (6.7%)	3 (17.6%)	0.603^b^
Delayed primary closure	8 (53.3%)	4 (23.5%)	0.082^b^
Delayed primary closure + STSG	3 (20%)	1 (5.9%)	0.319^b^
STSG	0	8 (47.1%)	0.003^b^
Local flap + STSG	2 (13.3%)	1 (6.3%)	0.589^b^
Free flap	1 (6.7%)	0 (17.6%)	0.469^b^
LOS(day), median (IQR)	13 (5–27)	26 (10–54)	0.002^c^
Outpatient follow-up in days, median (IQR)	15 (7–22)	36 (18–137)	< 0.001^c^

h: hour, LOS: length of hospital stay, STSG: split-thickness skin graft, NPWT: negative pressure wound therapy

^a^Chi-squared test

^b^Fisher’s exact test

^c^Mann–Whitney U test

## Discussion

Several factors affect the requirement of surgical intervention in patients with venomous snakebites. Suspicion of acute compartment syndrome; symptoms of local ecchymosis, skin necrosis, bullae or blisters, and fever; *Naja atra* envenomation; and ultrasound findings of absence of Doppler flow are predictors of the need for surgery in patients with snakebites.

The optimal timing and role of surgical intervention for the treatment of venomous snakebites remain controversial. Many studies have not recommended surgical intervention [3, 8, 19, 22, 23]; however, most of these studies were conducted in North America and focused on pit viper snakes. In a study conducted in India, Chattopadhyay et al. [24] reported that 24% of patients required surgical intervention. In South Korea, debridement was required in 46 of 111 patients (41.4%) with snakebites [18]. In another study conducted in South Korea [25], fasciotomy was required in 10.8% of patients who had an intracompartmental pressure of > 40 mmHg and symptoms of compartment syndrome. In other studies conducted in Taiwan, many patients with *Naja atra* envenomation required surgical intervention [8, 26, 27]. Treatments for venomous snakebites widely vary depending on the specific region and snake species. This study compared the outcomes of patients who underwent surgery within and after 24 h of being bitten. Our findings can help surgeons determine the timing for necessary interventions and the surgical procedures for patients with venomous snakebites.

The venom of *Naja atra*, a common snake species in Taiwan, consists of a cardiotoxin, neurotoxin, and hemotoxinin addition to phospholipase A2. The cardiotoxin is the most harmful to humans because it synergistically acts with phospholipase A2 to induce local tissue necrosis after snakebites [26, 28–30]. Surgical debridement of venomous snakebite cases reduces intracompartmental pressure, and the interstitial fluid and infectious pathogens can be drained and eradicated, respectively, through controlled tissue destruction. Su et al. [9] suggested that patients presenting with ecchymosis on the bite wound or requiring high antivenom doses are highly likely to require surgical intervention. Additionally, admission to the ICU, ventilator support, ionotropic support, and coagulative parameter abnormalities may be indicators of bite severity and therefore of the need for early surgical intervention. Recently, Lai et al. [10] reported that lower limb bites, limb swelling, bulla or blister formation, gastrointestinal effects, and fever are clinical predictors of surgery after *Naja atra* envenomation. This finding is consistent with that of our study. The timing of surgery may be affected by the availability of a medical facility and venom specialist, identification of snake species, patient’s response to antivenom, and observation period depending on the snake species.

In this study, the patients who underwent initial surgery after 24 h had a higher proportion of lower extremity bites and a lower incidence of suspected acute compartment syndrome; therefore, their clinical observation period was longer, and surgical intervention was not performed until more than 24 h after the bite. These patients required significantly more NPWT dressing foam changes, longer hospital stays, and more outpatient follow-up visits than those who underwent their initial surgery within 24 h.

Patients who require surgical intervention to reduce tissue swelling and necrosis and to control infection may benefit from early surgery. In such patients, early surgery can prevent the development of necrotizing fasciitis, preclude extensive tissue destruction beyond the bite site, and reduce the size of the surgical wound. We found that the patients who underwent surgery within 24 h had fewer overall bite-related surgeries and NPWT dressing foam changes, leading to shorter hospital stays and fewer outpatient follow-up visits. Moreover, earlier surgical intervention may be associated with a simple final reconstructive surgery. A larger study population is necessary to verify this finding. Surgical intervention is crucial for the treatment of venomous snakebites; hence, the identification of clinical predictors to support the surgeons’ decision of performing early surgical intervention is crucial for the management of venomous snakebites.

In several studies, sonography predominantly revealed swelling in the subcutaneous tissues after snakebites [31–34]. Sonography is a simple and noninvasive procedure to assess venom-related tissue injury. In our surgical group, 68.8% of the patients had a positive ultrasound finding. We also used vascular techniques to detect perfusion patterns in the affected local tissue. Ultrasound findings of absence of Doppler flow indicate insufficient vascular perfusion, which can cause local tissue necrosis. Early surgical intervention may mitigate the progression of edema, inflammation, and necrosis. A combination of physical examination and sonography may be beneficial to assess the severity and prognosis of snakebite envenomation. However, the ideal timing of the scan in relation to the time of bite is not standardized. A higher number of patients and more serial ultrasound examinations will be required to evaluate the progression of envenomation and determine the efficacy of sonography in assessing snakebites.

Negative pressure wound therapy (NPWT) is often used in chronic and acute wound care [35]. It can effectively drain local exudates and reduce local inflammatory reactions [36]. A combination of surgical intervention and NPWT for snake bites has been proven to be effective for controlling the release of inflammatory cytokines (interleukin-6, interleukin-10, and tumor necrosis factor-α) and alleviating systemic inflammatory reactions [37]. Significant limb swelling regression has also been noted [37]. In our surgical group that underwent initial surgery within 24 h, the use of NPWT reduced the need for subsequent complicated reconstructive surgery. Therefore, our treatment strategy is safe and effective.

True compartment syndrome after snakebites is rare [38–44]. The diagnosis of compartment syndrome with the indication of fasciotomy is based on the clinical findings of pallor, pulselessness, pain, color change in the fingers, and increased swelling in the affected area [3, 20, 21]. The presence of disproportional pain caused by passive flexion or extension of adjoining distal joints in the tightly swollen extremity is a key indicator (or perhaps the only indicator) that proceeding with fasciotomy is necessary rather than waiting for pulselessness or signs of paralysis. Measurement of intracompartmental pressure is not recommended when the diagnosis is clinically evident [20]. Most medical institutions in Taiwan do not have any intracompartmental pressure measurement equipment. When clinical findings suggest acute compartment syndrome and the patient fails to respond to adequate and prompt antivenom administration, fasciotomy or dermotomy may be appropriate. Follow-up combined with NPWT can reduce the comorbidity of patients with snakebites.

This study has several limitations that should be addressed. First, the small sample size limited the power of our study to detect significant differences. Second, the inclusion of a patient population from a single institution may have limited the generalizability of our findings. However, most surgeons involved in this study were trained at the same institute, thereby reducing variability in snakebite management at our institution. Third, because this retrospective study has certain selection biases, the results should be cautiously interpreted. Fourth, a comparison of the outcomes between early and late intervention groups for both upper and lower limbs may be beneficial and will be addressed in our future research. Finally, we focused on clinical symptoms to determine the timing of surgical intervention and did not compare the laboratory data between patients with and without surgical intervention. Moreover, the species of venomous snakes may vary throughout the island. Hence, we suggest modifying the treatment depending on the snake species and accessibility to antivenom.

## Conclusion

In patients with snakebites, advanced age, high-risk clinical manifestations (e.g., local ecchymosis and bulla or blister formation), *Naja atra* envenomation, and ultrasound findings of absence of Doppler flow are predictors of surgical intervention. Patients receiving surgical intervention within 24 h may require fewer overall bite-related surgeries and NPWT applications, leading to shorter hospital stays and fewer outpatient follow-up visits. Our findings can be helpful for patient management following snakebites. Additional studies with larger samples are warranted to support our findings.

## Data Availability

The data that support the findings of this study are available from the corresponding author, Chih-Sheng Lai, upon reasonable request.

## Abbreviations

h: Hour
LOS: Length of hospital stay
NPWT: Negative pressure wound therapy
STSG: Split-thickness skin graft
TCVGH: Taichung Veterans General Hospital
